# Red Grape By-Products from the Demarcated Douro Region: Chemical Analysis, Antioxidant Potential and Antimicrobial Activity against Food-Borne Pathogens

**DOI:** 10.3390/molecules29194708

**Published:** 2024-10-04

**Authors:** Adriana Silva, Raquel Martins, Vanessa Silva, Fátima Fernandes, Rosa Carvalho, Alfredo Aires, Gilberto Igrejas, Virgílio Falco, Patrícia Valentão, Patrícia Poeta

**Affiliations:** 1 Microbiology and Antibiotic Resistance Team (MicroART), Department of Veterinary Sciences, University of Traás-os-Montes and Alto Douro (UTAD), 5000-801 Vila Real, Portugal; adrianaa.silva95@gmail.com; 2 LAQV-REQUIMTE, Department of Chemistry, NOVA School of Science and Technology, Universidade Nova de Lisboa, 2829-516 Caparica, Portugal; 3 Department of Genetics and Biotechnology, University of Traás-os-Montes and Alto Douro (UTAD), 5000-801 Vila Real, Portugal; 4 Functional Genomics and Proteomics Unit, University of Traás-os-Montes and Alto Douro (UTAD), 5000-801 Vila Real, Portugal; 5 LAQV-REQUIMTE, Laboratoário de Farmacognosia, Departamento de Quiámica, Faculdade de Farmaácia, Universidade do Porto, 4050-313 Porto, Portugal; raquel2001martins@gmail.com (R.M.); mfgfernandes@gmail.com (F.F.); vfalco@utad.pt (V.F.); valentao@ff.up.pt (P.V.); 6 Department of Agronomy, School of Agrarian and Veterinary Sciences, University of Trás-os-Montes and Alto Douro (UTAD), 5001-801 Vila Real, Portugal; rpaula@utad.pt; 7 Centre for the Research and Technology of Agro-Environmental and Biological Sciences (CITAB), University of Traás-os-Montes and Alto Douro (UTAD), 5000-801 Vila Real, Portugal; alfredoa@utad.pt; 8 CECAV—Veterinary and Animal Research Centre, University of Traás-os-Montes and Alto Douro (UTAD), 5000-801 Vila Real, Portugal; 9 Veterinary and Animal Research Centre, Associate Laboratory for Animal and Veterinary Sciences (AL4AnimalS), University of Traás-os-Montes and Alto Douro (UTAD), 5000-801 Vila Real, Portugal

**Keywords:** winemaking, grape seeds, phenolic compounds, Gram-positive bacteria

## Abstract

Wine production is one of the most important agricultural activities. The winemaking process generates a considerable volume of different residues characterized as by-products, such as pomace, seeds, stems, and skins. By-products are rich in polyphenols with antioxidant and antibacterial properties and may act as bacteriostatic or bactericidal agents against food-borne pathogens, improving food safety by enhancing antibiotic efficacy and reducing bacterial resistance. The aim of this study was to evaluate the phenolic composition and antioxidant activity of grape components (skins, seeds, and stems) from three red grape varieties (Periquita, Gamay, and Donzelinho Tinto) and determine their antibacterial activity against antibiotic-resistant bacteria, including *Escherichia coli* in food-producing animals and *Listeria monocytogenes* from food products and food-related environments. Ten phenolic compounds were quantified in these red grape varieties, with specific compounds found in different parts of the grape, including phenolic acids and flavonoids. Flavonoids are abundant in seeds and stems, malvidin-3-*O*-glucoside being the main anthocyanin in skins. The ethanolic extract from the seeds showed in vitro concentration-dependent activity against reactive species like ^•^NO and O_2_^•−^. Gamay extract was the most effective, followed by Donzelinho Tinto and Periquita. Extracts showed varying antibacterial activity against Gram-positive and Gram-negative bacteria, with stronger effects on Gram-positive bacteria. *L. monocytogenes* was more susceptible, while *E. coli* was limited to three strains. Seeds exhibited the strongest antibacterial activity, followed by stems. The results of our study provide evidence of the potential of grape by-products, particularly seeds, as sources of bioactive compounds with antioxidant and antibacterial properties, offering promising avenues for enhancing food safety and combating antibiotic resistance in food production and related environments.

## 1. Introduction

Wine production is one of the most important agricultural activities in the world, and grapes are highly valued as food. As one of the world’s largest fruit crops, more than 60 million tons of grapes are produced annually, and 30% of the total volume of grapes vinified produces wine by-products, equivalent to almost 20 million tons, and 50% of the by-products in the European Union [1].

The wine industry contributes significantly to global environmental problems, such as pollution and soil degradation [2]. The production and non-utilization of residues and by-products like pomace, seeds, stems, and skins are problems leading to a loss of matter and energy, with a negative impact on nature and health [3]. Although these by-products are non-toxic, their high organic content and seasonal production generate large amounts of waste and loss of valuable materials in the industry. Efforts have been made to reuse by-products in other parts of the production chain [3,4].

To contribute to a healthy environment, waste from the wine industry is processed using mechanical, chemical, or biological methods and used as raw material for new products and applications. This approach, known as the circular economy, aims to create a “zero waste” society [4]. The 12th Sustainable Development Goal (SDG) addresses this issue and focuses on sustainable consumption and production. The report highlights the need to strengthen the scientific and technological capacity to implement sustainable and circular production and consumption models through avoidance, reduction, recycling and reuse [5]. In fact, the United Nations’ 2030 Agenda for Sustainable Development calls for adding value to by-products by reducing post-harvest losses, moving them to processing and retail, and recovering bioactive compounds from food processing residues [6]. It emphasizes the need and the urgency of sustainability in the wine industry and has shifted research attention to examining the management of wine by-products from a different perspective [3]. These by-products and individual components of grapes are residues produced during the pressing of red and white grapes and are an important source of added-value compounds, mainly flavonoids, phenolic acids, and other phenolic compounds, such as stilbenes [3]. Phenolic compounds, a diverse group of secondary metabolites found in plants, possess great biological importance due to their various physiological and health-promoting properties. These compounds are known for their strong antioxidant activity, which helps to neutralize free radicals and prevent oxidative stress-related damage in cells. They also have significant effects against bacteria by causing structural or functional damage to bacterial cell membranes. They target various bacterial cell components, such as the cell wall, cell membrane, bacterial proteins, and adhesive structures and play a crucial role in plant defense mechanisms against pathogens and environmental stressors. Additionally, phenolic compounds exhibit anti-inflammatory, anti-carcinogenic, and cardioprotective effects [7,8,9,10]. Due to their numerous properties, grape and wine by-products are currently used in various fields like agriculture, cosmetics, pharmaceuticals, biorefineries, animal feed, and the food industry [3], constituting opportunities for economic transformation that ensures increased sustainability while reducing the ecological footprint [4].

Thus, the aim of this study was to elucidate the phenolic composition of various individual vegetal materials of grapes (skins, seeds, and stems) and determine their antibacterial activity against several antibiotic-resistant bacteria, including *Escherichia coli*, in food animals (such as pigs and rabbits), and *Listeria monocytogenes* from food and food-related environments. Additionally, this study investigated the antioxidant activity of these grape individual components using various biological assays. The grapes studied were from three red varieties used in the Demarcated Douro region: Periquita, Gamay, and Donzelinho Tinto.

## 2. Results

### 2.1. Phenolic Compounds in Hydroethanolic Extracts

**Figure 1:**
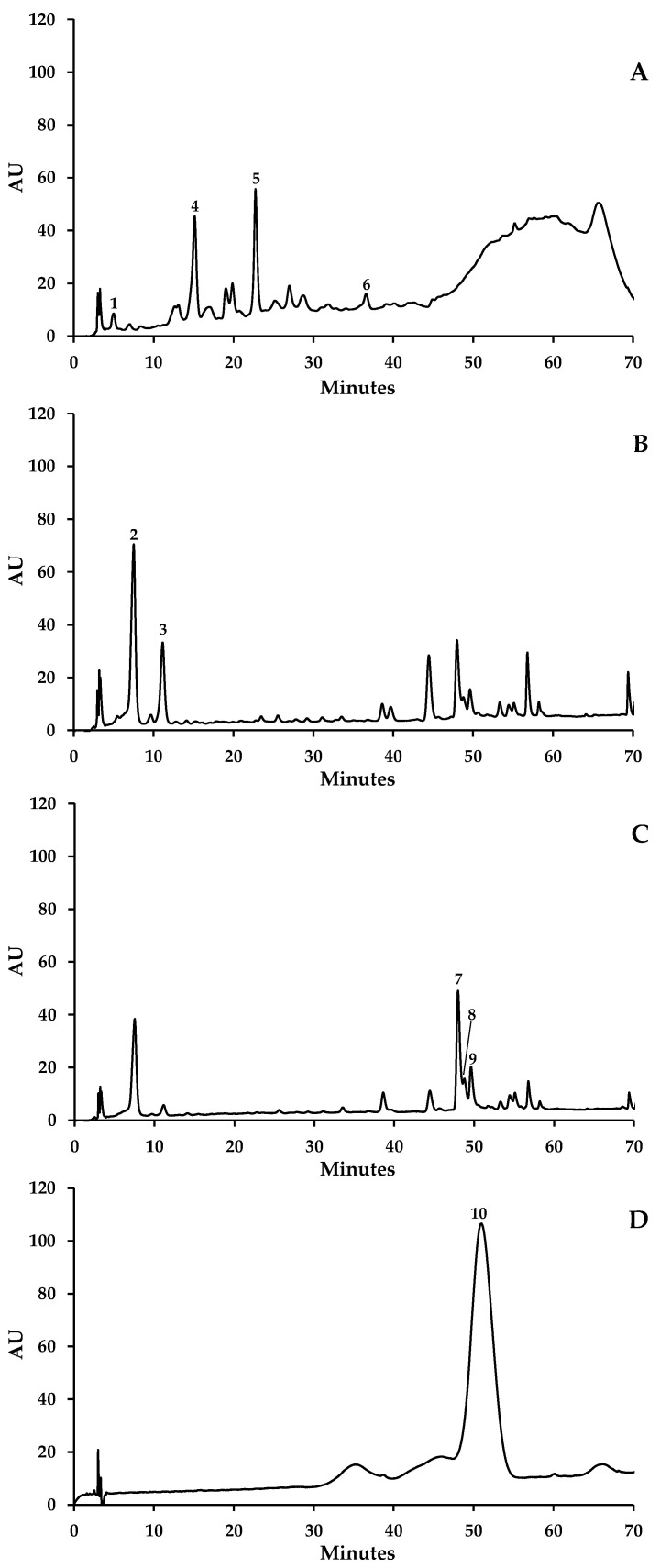
Representative HPLC-DAD chromatogram of the phenolic profile of hydroethanolic extracts obtained from (**A**) seeds (detection at 280 nm), (**B**,**C**) stems (detection at 320 and 350 nm, respectively), and (**D**) skins (detection at 500 nm) of Periquita grapes. Gallic acid (**1**), caftaric acid (**2**), coutaric acid (**3**), catechin (**4**), epicatechin (**5**), epicatechin-3-*O*-gallate (**6**), quercetin-3-*O*-galactoside (**7**), quercetin-3-*O*-rutinoside (**8**), quercetin-3-*O*-glucoside (**9**), and malvidin-3-*O*-glucoside (**10**).

Eleven phenolic compounds distributed by phenolic acids (gallic (**1**), caftaric (**2**) and coutaric acids (**3**)), flavan-3-ols (catechin (**4**), epicatechin (**5**), epicatechin-3-*O*-gallate (**6**)), flavonols (quercetin-3-*O*-galactoside (**7**), quercetin-3-*O*-rutinoside (**8**), quercetin-3-*O*-glucoside (**9**) and kaempferol-3-*O*-glucoside (**11**)) and one anthocyanin (malvidin-3-*O*-glucoside (**10**)) were identified in the three different vegetal materials and varieties of red grapes (Figure 1). Only compounds for which complete identification was achieved (Figure 1) were quantified. They are listed in Table 1.

Distinct profiles were found among the various plant tissues and grape varieties. No phenolic acid was detected in the skins. Seeds were the only vegetal material that contained a hydroxybenzoic derivative, gallic acid (**1**). The content of gallic acid (**1**) was similar among the three grape varieties (0.24, 0.30, and 0.31 mg/g dry samples in Periquita, Gamay, and Donzelinho Tinto, respectively) (Table 1). On the other hand, caftaric (**2**) and coutaric (**3**) acids were only obtained from stem extracts; the first one was predominant in all grape varieties (Table 1).

The skins also did not show flavan-3-ols in their composition. Catechin (**4**) was the only flavan-3-ol whose presence was common to the seeds and stems of all three grape varieties, being the most representative compound of this flavonoid subclass. Epicatechin-3-*O*-gallate (**6**) was the minor flavan-3-ol found, with only trace amounts being detected in seed extracts. The hydroethanolic extract obtained from Gamay variety seeds was the richest in these compounds (12.92 mg/g dry sample), followed by hydroethanolic extracts of Periquita seeds and Donzelinho Tinto stems, with about half the content (6.77 and 6.74 mg/g dry sample, respectively) (Table 1). On the other hand, flavonols were only detected in skin and stem extracts. Only skin extracts exhibited the four identified compounds, quercetin-3-*O*-galactoside (**7**), quercetin-3-*O*-rutinoside (**8**), quercetin-3-*O*-glucoside (**9**), and kaempferol-3-*O*-glucoside (**11**), with the last one being not detected in the stem extracts. This material presented smaller quantities of this kind of compound than the skin extracts (Table 1).

Malvidin-3-*O*-glucoside (**10**), the only anthocyanin identified, was only found in the extracts of the skins and stems, with the exception of the extract obtained from the stems of Donzelinho Tinto, in which this compound was not found. The skins were the material with a higher content of malvidin-3-*O*-glucoside (**10**).

Overall, the extracts with the highest total phenolic content were obtained from the skins and followed the order Donzelinho Tinto > Periquita > Gamay varieties.

### 2.2. Antioxidant Activity

The screening of the antioxidant activity of the hydroethanolic extracts obtained from skins, seeds, and stems of Periquita, Gamay and Donzelinho Tinto grape varieties was performed using the DPPH method. The DPPH method has been widely used to determine antioxidant free radical scavenging capacity [10], providing initial information about the antioxidant potential of a compound, an extract, or other biological source [11]. So, it was conducted to identify and select those extracts with the strongest antioxidant activity for subsequent biological tests. As shown in Table 2, the seed extracts exhibited the lowest IC_50_ values. Therefore, they were selected for the subsequent ^•^NO and O_2_^•−^ scavenging tests.

**Figure 2:**
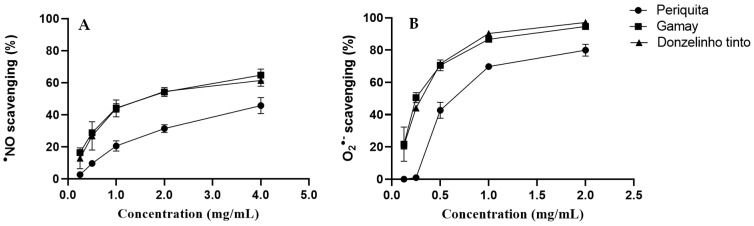
^•^NO (**A**) and O_2_^•−^ scavenging (**B**) activity by red grape seed hydroethanolic extract. Results are expressed as mean ± SD of three independent experiments, each performed in triplicate.

The hydroethanolic extracts obtained from the three different varieties of seeds were able to intercept the in vitro-generated reactive species ^•^NO and O_2_^•−^ in a concentration-dependent way (Figure 2).

The positive control used for both ^•^NO and O_2_^•−^ assays was quercetin (IC_25_ = 0.007 ± 0.001 mg/mL and IC_25_ = 0.024± 0.003 mg/mL, respectively). In the ^•^NO assay, the Periquita seed extract was unable to reach 50% activity. Consequently, IC_25_ values were determined to compare the activity of all extracts. For the different seed extracts, the IC_25_ values determined against O_2_^•−^ were lower than those observed for ^•^NO. The IC_25_ values found against ^•^NO were 1.31 ± 0.19 mg/mL with Periquita, 0.47 ± 0.13 mg/mL with Donzelinho Tinto, and 0.42 ± 0.03 mg/mL with Gamay extract. The same order of scavenging activity was noticed against O_2_^•−^: 0.38 ± 0.03 mg/mL with Periquita, 0.14 ± 0.01 mg/mL with Donzelinho Tinto, and 0.13 ± 0.00 mg/mL with Gamay extract. Thus, the Gamay variety extract was the most effective in both tests, followed by Donzelinho Tinto and Periquita extracts.

### 2.3. Antimicrobial Activity

The antimicrobial activity of skin, seed, and stem extracts from the three red grape varieties was evaluated using the Kirby–Bauer disc diffusion method. The results for the minimum inhibitory concentration (MIC) are expressed in Table 3. As observed, the results are different when comparing the activity against Gram-positive and Gram-negative bacteria, and there are clear differences between the two. Among the Gram-negative bacteria, only three strains of *E. coli*, isolated from samples from pig farms, demonstrated sensitivity to the extracts studied, except for extracts obtained from skins. Strain S1 was susceptible to all seed and stem extracts, with the best antimicrobial activity being observed with the seed extract and with the Gamay stem extract (MIC = 10 μg/mL). Strain S18 was just susceptible to Periquita and Gamay seed extracts, the last being the most effective (MIC = 25 μg/mL), while antimicrobial activity against strain S34 was revealed only by Donzelinho Tinto stem extract (MIC = 75 μg/mL). The *E. coli* strains isolated from rabbit farms were resistant to all of the extracts studied. Concerning Gram-positive bacteria, all extracts revealed antimicrobial effects against *L. monocytogenes*, except the one obtained from the skins of the Gamay variety. In general, this bacterium was more susceptible to seed extracts, as evidenced by the lower MIC values found. Regarding extracts from grape stems and skins, the first was found to be more effective against Gram-positive bacteria than skins.

## 3. Discussion

The agri-food industry generates large amounts of residues and is considered to be the second largest producer of waste in the environment [12], posing environmental challenges and causing significant economic losses. In the wine industry, by-products like grape seeds, skins, and stems can be converted into valuable bioactive substances, especially phenolic compounds [12,13]. These secondary metabolic compounds are characterized by having health-promoting properties. Therefore, there is an increasing interest in the evaluation and utilization of by-products generated during the different stages of wine production due to their availability, low cost, and sustainability as sources of a broad range of bioactive compounds [7,14].

The phenolic profiles of the distinct grape materials of the three red grape varieties herein established comprise phenolic acids, flavan-3-ols, flavonols, and anthocyanins, with qualitative (Figure 1) and quantitative differences (Table 2) being noticed. None of the three grape tissues analyzed presented the four subclasses of phenolic compounds found herein (Table 2). The skins were qualitatively the poorest material due to the absence of phenolic acids and flavan-3-ols, though they contained the highest amount of phenolic compounds, mainly due to the high malvidin-3-*O*-glucoside (**10**) content, which was similar among the three varieties (between c.a. 39 and 42 mg/dry sample in the skins) (Table 1). Malvidin-*3-O*-glucoside (**10**), belonging to the reddest anthocyanins due to the high degree of methylation of its B ring, is described as the main anthocyanin in red grapes [12,14]. In a general way, anthocyanins are reported to be the main compounds found in the skins of red grape varieties. Although it was also present in the extracts obtained from the stems of all varieties herein studied, tmalvidin-3-*O*-glucoside (**10**) content in this grape material was around 50× lower than that found in the skins (Table 1).

Anthocyanins, synthesized via the flavonoid pathway, are crucial phenolic compounds responsible for the red color of grapes and wine. As the most important natural colorants in grapes and their products, anthocyanins play important roles in plants, including protection against radiation (solar exposure and ultraviolet radiation), defense against pathogens, attraction of predators for seed dispersal, and the modulation of signaling pathways [15]. These natural pigments also possess known pharmacological properties and are used by humans for therapeutic purposes [16]. A previous study focusing on Italian red wine showed that the anthocyanin fraction was the most effective both at scavenging reactive oxygen species and inhibiting lipoprotein oxidation and platelet aggregation, suggesting that anthocyanins could be the key component in red wine that protects against cardiovascular disease. Anthocyanins are also characterized by their potential use as anti-inflammatory and anti-edema, antimutagenic, hepatoprotective, and tumor cell growth-suppressing agents [16]. Malvidin-3-*O*-glucoside, in particular, has already been revealed to have the capacity to inhibit human macrophage-derived inflammatory mediators and decrease clinical scores in arthritic rats. Furthermore, its low toxicity compared to most current immunosuppressor agents has raised interest in the use of this compound for therapeutics of Th1/macrophage-dominant inflammatory diseases like rheumatoid arthritis [17].

Concerning phenolic acids, gallic acid (**1**) was only detected in the seeds, and caftaric (**2**) and coutaric (**3**) acids were only found in the stems. The first has been indicated before as being an anti-Alzheimer agent [18,19]. Moreover, several cellular and animal studies have reported on the numerous health-promoting effects of gallic acid, including its antioxidant, antimicrobial and anticancer properties, and it also plays a very promising role in gastrointestinal, cardiovascular, and metabolic disease prevention [19].

The predominance of caftaric acid (**2**) in all grape varieties is in accordance with the literature, with this compound being the predominant phenolic acid in both *Vitis vinifera* L. and non-vinifera types [20]. Correlation studies have shown a direct relationship between the content of caftaric and coutaric acids and the scavenging activity against free radicals exhibited by polyphenol-rich extracts obtained from grapes and their resulting products [21].

Flavan-3-ols, known components of grapes, play a significant role in grape materials, influencing various aspects, from health benefits to wine production [22]. They are mainly responsible for the color, taste, mouth feel, oxidation, and other chemical reactions [23]. In our study, flavan-3-ols were found in high concentrations in grape seeds, with them being the most abundant phenolic compounds in this material. Furthermore, they play an important role as sensory components that contribute to wine’s bitterness and astringency. Furthermore, they are responsible for browning reactions in grapes and wine [23].

Flavonols are found in grape skins and stems, and hydroxycinnamic acids were identified as the main groups of UV-absorbing phenolics [24]. Flavonol synthesis is significantly stimulated by natural UV, in particular UV-B. The acclimation of UV screening depends almost exclusively on flavonol synthesis [24]. The monomers catechin (**4**) and epicatechin (**5**) are commonly found in grapes at similar levels or the amounts of epicatechin (**5**) are higher; our results show a slight predominance of catechin (**4**), both in seeds and stems.

Besides their significant impact on the sensory attributes of wine, catechins and epicatechins are powerful antioxidants [25], exhibiting potent radical-scavenging ability, which protects proteins, nucleic acids, lipids, and carbohydrates from damage caused by reactive oxidative species. They also have the capacity to prevent lipid peroxidation, a process that can lead to cell membrane damage and is implicated in various diseases [26,27]. Looking at the results obtained from the DPPH assay, flavan-3-ols seem to be the major contributors to the strongest antiradical activity observed for the extracts obtained from grape seeds (Figure 2 and Table 1). Given the biological relevance of nitric oxide (^•^NO) and superoxide radical anion (O_2_^•−^) in the human body [28], the ability of seed extracts to scavenge these two radicals was also studied.

The ethanolic extracts of the seed grapes showed a concentration-dependent ^•^NO and O_2_^•−^ scavenging capacity, with the Periquita variety being less potent for both radicals. These results can be explained by the phenolic composition of the distinct grape varieties. In fact, among the three grape seed varieties tested, the Gamay seed extract was the one that revealed the highest total phenolic content (13.23 ± 0.07 mg/mL). Although the highest content of catechins can contribute to these results, other phenolic compounds will certainly contribute to the activity observed since Periquita, the second variety with the highest flavan-3-ols content, was not the second-most active sample (Figure 2, Table 1). In summary, although the antiradical activity exhibited by the grape seed extracts points to the interference of phenolic compounds, it should also be noted that extracts obtained from natura matrices are complex mixtures; other non-determined compounds could also contribute to the observed activity.

The antibacterial activity of by-products from different red grape varieties from the Douro region was evaluated, and it was found that Gram-negative bacteria were less susceptible to these extracts than Gram-positive bacteria. Food-borne bacteria, including *E. coli* isolated from livestock and *L. monocytogenes* isolated from food and associated environments were used in the study. Among the by-products tested, seed extracts showed the strongest antibacterial activity against these pathogens, followed by extracts from the stems and skin. In general, the antibacterial potential of extracts from winemaking by-products has already been reported and confirmed in other studies. It has been shown that seed extracts from Portuguese red grape varieties, such as Touriga Nacional and Preto Martinho [29], are more effective against Gram-positive bacteria than Gram-negative bacteria. This result was observed not only for Portuguese varieties but also in studies carried out in the USA, India, Turkey, and New Zealand [1]. This occurs because Gram-negative bacteria have a double-layered cell wall membrane, the outer membrane of which potentially prevents the uptake of phenols from the extract [30]. As a result, Gram-negative bacteria are more resistant to the antibacterial effects of the extracts compared to Gram-positive bacteria. In contrast to Gram-negative bacteria, Gram-positive bacteria have only one barrier, allowing lipophilic compounds to penetrate more easily [31,32]. However, it is also described that the physicochemical characteristic of the extracts is the main factor for the distinct properties, followed by the structural difference in the bacteria [32].

Grape seed extracts have demonstrated antimicrobial activity against food-borne pathogens, such as *E. coli* and *L. monocytogenes*, with particularly strong effectiveness against Gram-positive bacteria. Many scientific works report that grape seeds contain greater amounts of total phenolic compounds when compared to skins and stems [32]. The major groups of compounds that are responsible for antimicrobial activity include phenolic acids, quinones, saponins, flavonoids, tannins, coumarins, terpenoids, and alkaloids [33]. The extracts obtained from the seeds of the different grape varieties studied herein showed phenolic acids and flavonoids in their composition, with the Gamay seed extract being the richest in such compounds, followed by Periquita and Donzelino Tinto. This is in line with the results of the antibacterial and antioxidant activities, where Gamay demonstrated better results in all parameters. Although the effect of an extract cannot be simply extrapolated from the activities of their isolated compounds, the synergic effects between catechin (**4**), epicatechin (**5**), and gallic acid (**1**) can be suggested as being additional factors for the differential antibacterial activity observed with grape seeds extract [30]. These compounds generally show greater activity against Gram-positive bacteria; however, in this study, it was possible to observe activity against Gram-negative bacteria from three strains of *E. coli* isolated from pigs. These results may be influenced by a variety of factors, including the strong correlation between grape polyphenol levels and the growth of Gram-negative bacteria [34], the grape variety, climatic conditions, the extraction solvent, and the specific bacterial species involved [35].

Grape seed by-products contain phytochemicals that act as antioxidants, antibacterial agents, and health-promoting agents; therefore, they have been used in livestock feed in various studies [36]. Grape processing waste extracts can be used as natural preservatives in meat products since studies have shown their efficiency in inhibiting the growth of microorganisms related to outbreaks of food-borne diseases, as verified in our study [37]. Due to their antimicrobial activity, winemaking by-product extracts could be a valuable tool in combating one of the biggest public health challenges of the 21st century: antibiotic resistance [1]. Additionally, they address the pressing need to ensure the environmental sustainability of agricultural production. The positive effect on animal growth is due to the presence of nutrients and bioactive compounds that improve gut health. However, the inclusion of high amounts of grape by-products in livestock feed can affect growth and reduce the effect of spoilage caused by the multiplication of microorganisms or chemical reactions during the storage period [36,38]. As observed in other studies and our study, strategies using wine by-products have gained interest as they provide antimicrobial benefits that improve the health and safety of food animals and contribute to sustainable, drug-free agricultural systems [39]. The need to minimize the environmental impact of waste disposal is increasing the value of industrial grape by-products. As consumer access to a variety of foods increases worldwide, the demand for food preservation methods is growing [40]. Sustainable wine production practices are growing in popularity, and the food industry and stakeholders prioritize environmental concerns and adopt green technologies [37,39].

**Figure 3:**
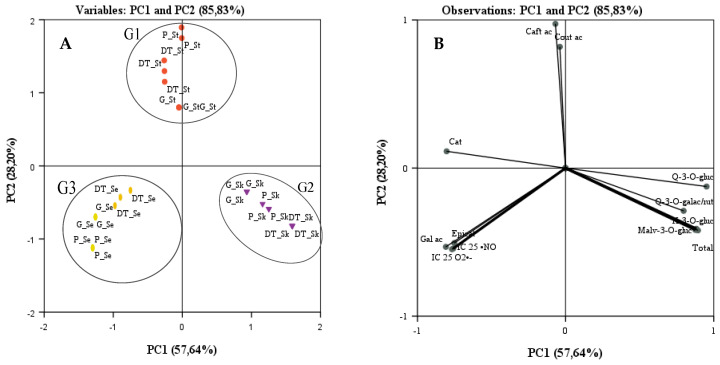
Projection of grape by-products (skins, seeds, and stems) of Donzelinho Tinto, Gamay, and Periquita varieties (**A**) (variables: Gamay skins (G_Sk), Gamay seeds (G_Se), Gamay stems (G_St), Periquita skins (P_Sk), Periquita seeds (P_Se), Periquita stems (P_St), Donzelinho Tinto skins (DT_Sk), Donzelinho Tinto seeds (DT_Se), and Donzelinho Tinto stems (DT_St) and loadings by (**B**) phenolic compounds composition (variables: gallic acid (Gal ac); caftaric acid (Caft ac); coutaric acid (Cout ac); catechin (Cat); epicatechin (Epicat); epicatechin gallate (Epicat dev); quercetin-3-*O*-galactoside (Q-3-*O*-gal); quercetin-3-*O*-glucoside+quercetin-3-*O*-rutinoside (Q-3-*O*-glut+Q-3-*O*-rut), kaempferol-3-*O*-glucoside (K-3-*O*-glu); malvidin-3-*O*-glucoside (Malv-3-*O*-glu); IC_25_ values obtained for O_2_^•−^ assay (IC_25_ O_2_^•−^); IC_25_ values obtained for ^•^NO assay (IC_25_^•^NO) into the plane composed by the principal components PC1 and PC2 containing 85.83% of the total variance for phenolic compounds and IC_25_ values.

To study the relationship between the by-products of different red grapes varieties and their phenolic composition and antioxidant activity, PCA was performed, considering the content (mg/Kg dry sample) of phenolic compounds and IC_25_ values for O_2_^•−^and ^•^NO assays (Figure 3).

PCA for all datasets explained between 85.83% of the total variance; PC1 accounts accounting for 57.64% of the variance, and PC2 accounts for 28.20% (Figure 3). As shown in Figure 3A, three groups can be clearly distinguished.

One group (G1) includes the stems of the three grape varieties analyzed, Donzelinho Tinto, Gamay, and Periquita, as characterized by red dots. These by-products appeared on the positive plan of PC2 because they are the only grape components that contain caftaric (**2**) and coutaric (**3**) acids, with the presence of catechin (**4**), as major flavan-3-ols, also contributing to the placement of this group (Figure 3A,B). Within the G1 group, it was verified that the Periquita stems were positioned in the most positive part of PC2 because it is the variety richest in the above-mentioned hydroxycinnamic acids and the poorest in catechin (**4**) (Figure 3A,B).

G2, marked with yellow dots, located on the positive plan of PC1 and negative plan of PC2, included all skin varieties due to their high amounts of flavonols, malvidin-3-*O*-glucoside (**10**), and total phenolic content (Figure 3A,B). On the other hand, the absence of flavonols and malvidin-3-*O*-glucoside (**10**), their high catechin (**4**), and epicatechin (**5**) content, as well as the presence of gallic acid (**1**) only in seeds, led to the inclusion of these grape materials in another group (G3). G3 is characterized by purple dots appearing in the negative part of PC1, clearly distinguishing the G2 group; furthermore, the best antiradical activity was observed for these samples (Figure 3A,B).

## 4. Materials and Methods

### 4.1. Standards and Reagents

Catechin, epicatechin, and epigallocatechin-3-*O*-gallate were from Extrasynthese (Genay Cedex, France). Gallic acid, caffeic acid, *p*-coumaric acid, quercetin-3-*O*-rutinoside, kaempferol-3-*O*-glucoside, malvidin-3-*O*-glucoside, quercetin-3-*O*-glucoside, quercetin, 2,2-diphenyl-1-picrylhydrazyl (DPPH), sodium nitroprusside dihydrate (SNP), sodium phosphate, phosphoric acid (H_3_PO_4_), sulphanilamide, β-nicotinamide adenine dinucleotide reduced form (NADH), nitroblue tetrazolium (NBT), phenazine methosulphate (PMS), and monopotassium phosphate (KH_2_PO_4_) were acquired from Sigma-Aldrich (St. Louis, MO, USA). N-(1-naphthyl) ethylenediamine dihydrochloride was obtained from Fisher chemical (UK). Acetic acid, acetonitrile, and methanol (MeOH) were purchased from Merck (Darmstadt, Germany). Water was treated in a Milli-Q water purification system (Millipore, Bedford, MA, USA).

### 4.2. Plant Material and Samples Preparation

The plant material used consisted of fresh fruits from different red grape varieties, namely Periquita, Gamay, and Donzelinho Tinto. Grapes were collected from healthy plants in the experimental vineyard of the University of Trás-os-Montes and Alto Douro, Vila Real (41°190 N, 7°440 W, 500 m above mean sea level) of the Baixo Corgo sub-region of the Demarcated Douro Region, northern Portugal, during the harvesting season in September. The seeds, skins, and stems of grapes were manually separated and then lyophilized. The dried material was powdered in an appliance mill (model A327R1, Moulinex, Spain). The powdered material (≤910 µm) was kept in a desiccator in the dark until analysis.

Voucher specimens were deposited at Laboratório de Farmacognosia, Faculdade de Farmácia, Universidade do Porto, and at Laboratório de Marcadores Moleculares, University of Trás-os-Montes and Alto Douro under the following designations: P_Se_2022 (Periquita seeds), P_Sk_2022 (Periquita skins), P_St_2022 (Periquita stems), G_Se_ 2020 (Gamay seeds), G_Sk_ 2020 (Gamay skins), G_St_ 2020 (Gamay stems), DT _Se_2022 (Donzelinho Tinto seeds), DT _Sk_2022 (Donzelinho Tinto skins), and DT _St_2022 (Donzelinho Tinto stems).

### 4.3. Extraction of Phenolic Compounds

The phenolic constituents from the grape skins, seeds and stems were extracted using an ethanol/water (80:20) (*v*/*v*) mixture. For each case, 0.5 g of powdered sample was mixed with 50 mL of the ethanol/water mixture under stirring for 1 h. The extract was centrifuged (10 min and 4000 rpm), and the pellet was re-extracted. The combined supernatants were collected, and the solvent ﻿was evaporated to dryness under reduced pressure at 40 °C. For the phenolic characterization and screening of antioxidant activity using the DPPH assay, the grape skin, seed, and stem extracts were redissolved in absolute ethanol. For the antimicrobial activity and other biological assays, the dry residues were weighed and redissolved in dimethyl sulfoxide (DMSO).

### 4.4. HPLC-DAD Analysis of Phenolic Compounds

For phenolic compounds characterization, an aliquot of 20 µL of each extract, previously filtered through a 0.45 µm size pore membrane, was injected into a Gilson HPLC-DAD unit equipped with a Spherisorb ODS2 column (25.0 cm × 0.46 cm, 5 um particle size; Waters, Milford, MA, USA), following the chromatographic conditions previously described by Ferreira et al. [41]. Elution was performed with 2% (*v*/*v*) acetic acid in water (eluent A) and a solution of 0.5% (*v*/*v*) acetic acid in water and acetonitrile (50:50, *v*/*v*, eluent B), using a gradient program as follows: from 10% to 24% B (20 min), from 24% to 30% B (20 min), from 30% to 55% B (20 min), from 55% to 70% B (5 min), from 70% to 80% B (5 min), from 80% to 100% (5 min), and 100% B isocratic (5 min). The flow rate was 1.0 mL/min. Chromatograms were registered at 280 nm for hydroxybenzoic acids and flavan-3-ols, 320 nm for hydroxycinnamic acids, 350 nm for flavonols, and 500 nm for anthocyanins.

Caftaric (**2**) and coutaric (**3**) acids were identified by comparing their retention times and UV-Vis spectra with compounds previously identified by our group in wine samples by using the same methodology [42]. The other compounds were identified by comparing their retention times and UV spectra with those of authentic standards. Quantification was performed using the external standard method. The linearity range of the method was assessed by building calibration curves using five different concentration levels of the pure standards according to the range of concentrations present in the samples (Table 4). Caftaric (**2**) and coutaric (**3**) acids were determined as *p*-coumaric acid and caffeic acid, respectively. Epicatechin-3-*O*-gallate (**6**) was quantified as epigallocatechin-3-*O*-gallate and quercetin-3-*O*-galactoside (**7**) and quercetin-3-*O*-rutinoside (**8**) were quantified together as quercetin-3-*O*-rutinoside. The other compounds were determined as being themselves. The samples and standards were analyzed in triplicate.

### 4.5. Antioxidant Activity and Biological Assays

#### 4.5.1. DPPH^•^ Scavenging

The antiradical activity of the skin, stem, and seed extracts was determined according to Barbosa et al. [43]. In this assay, 25 μL of serial dilutions of the extract were mixed with 200 μL DPPH solution (150 µM), followed by 30 min incubation in the dark at room temperature. The absorbance was read at 515 nm. The scavenging capacity was calculated according to the following equation: DPPH^•^ scavenging (%) = 100 × [1 − (A extract − A blank)/(A control − A blank)]. A corresponds to the absorbance measured. Three independent experiments were performed, each one in triplicate. Ascorbic acid was used as the positive control.

#### 4.5.2. Superoxide Anion Radical Scavenging

The capacity of the seed hydroethanolic extracts to scavenge superoxide anion radical (O_2_^•^) was determined using a non-enzymatic system (NADH/PMS system, at PH 7.4) based on the reduction of nitroblue tetrazolium (NBT) to nitroblue diformazan, which can be spectrophotometrically determined, as described by Lopes et al. [44]. Then, 50 μL of serial dilutions of extract were mixed with 50 μL of NADH solution (166 μM) and 150 μL of NBT solution (43 μM). The reaction started after the addition of 50 μL of PMS solution (2.7 μM), and the absorbance was read at 560 nm. The antiradical capacity was calculated using the following equation: O_2_^•−^ scavenging (%) = 100 × [1 − (A extract/A control)]. Here, A corresponds to the absorbance measured. Three independent experiments, each performed in triplicate, were assayed. Quercetin was used as the positive control.

#### 4.5.3. Nitric Oxide Radical Scavenging

The capacity of the seed hydroethanolic extracts to scavenge nitric oxide radicals (^•^NO) was determined via the Griess reaction, according to Pereira et al. [45]. In the 96-well plate, 100 μL of SNP (20 mM) and 100 μL of a serial dilution of each extract were incubated for 60 min at room temperature under white light exposure. Afterward, the same volume (100 μL) of Griess reagent (1% sulphanilamide and 0.1% N-(1-napthyl) ethylenediamine in 2% H_3_PO_4_)) or phosphoric acid (H_3_PO_4_) (2%) (for blank) was added to each well and incubated at room temperature for 10 min in the dark. After that, the absorbance was read at 562 nm. The scavenging of nitric oxide radicals was calculated according to the following equation: ^•^NO scavenging (%) = 100 × [1 − (A extract − A blank)/(A control − A blank)]. Here, A corresponds to the absorbance measured. Three independent experiments were performed, each one performed in triplicate. Quercetin was used as the positive control.

#### 4.5.4. Data Processing and Statistical Analysis

Data analysis was performed using GraphPad Prism 8 Software, Inc. (San Diego, CA, USA) for Windows. Principal component analysis (PCA) was carried out using SPSS 21.0 software (IBM, NY, USA). PCA was applied to reduce the number of variables (12 variables corresponding to each identified phenolic compound (gallic acid (Gal ac), caftaric acid (Caft ac), coutaric acid (Cout ac), catechin (Cat), epicatechin (Epicat), epicatechin gallate (Epicat gal), quercetin-3-*O*-glucoside (Q-3-*O*-gluc), quercetin-3-*O*-galactoside+quercetin-3-*O*-rutinoside (Q-3-*O*-galac/rut), kaempferol-3-*O*-glucoside (K-3-*O*-gluc), and malvidin-3-*O*-glucoside (Malv-3-*O*-gluc)), IC_25_ O_2_^•−^ and IC_25_ ^•^NO) to derived variables (principal components: PCs) that adequately summarize the original information, i.e., the phenolic composition of the different grape by-products (skins, seeds, and stems). PCA shows similarities between samples projected on a plan and makes it possible to identify the variables that determine these similarities and in what way.

#### 4.5.5. Antibacterial Activity against Food-Borne Pathogens

Antimicrobial susceptibility was tested against 39 food-borne bacteria: 13 *Escherichia coli* (*E. coli*) isolated from healthy pigs farms [46], 13 *Escherichia coli* isolated from healthy rabbits farms [47], and 13 *Listeria monocytogenes* (*L. monocytogenes*) isolated from food products and food-associated environments [48]. All of the bacterial strains were grown in BHI agar (Oxoid, UK) for 24 h at 37 °C. For the antimicrobial activity assay, Müller–Hinton (Oxoid, UK) agar was used under the same conditions as in the previous series. The Müller–Hinton plates were inoculated with a swab dipped into a bacterial suspension with a turbidity equivalent to 0.5 McFarland standard. The antimicrobial susceptibility assay was performed using the Kirby–Bauer disc diffusion method. The initial extract solution of 100 μg/mL was diluted with DMSO to 75, 50, 25, and 10 μg/mL. Twenty microliters of the extracts in several concentrations was loaded on sterile blank discs (6 mm diameter), and the discs were impregnated onto inoculated agar. Discs with antibiotics were used as positive controls, and discs impregnated with DMSO were used as negative controls. The plates were incubated at 37 °C for 18–24 h. The inhibition zones indicated the antimicrobial activity of the extracts, which were measured with a ruler. The test was performed in triplicate.

## 5. Conclusions

The results obtained herein provide evidence of the antimicrobial and antioxidant activity of grape by-products. Flavonoids are abundant in seeds and stems, and seed extracts demonstrate concentration-dependent antioxidant activity against reactive species with biological significance. The extracts exhibited different levels of antibacterial activity, revealing their effectiveness against Gram-positive bacteria. Overall, grape seeds seem to be a promising natural solution to improving food safety and the sustainability of agricultural production systems. The exploitation of this material would reduce the environmental impact caused by the winemaking industry while also resulting in economic advantages and addressing public health challenges.

## Figures and Tables

**Table 1:** Phenolic compounds in hydroethanolic extracts of red grape material (mg/g dry sample).

Phenolic Compounds	Retention Time (min)	Seeds			Skins			Stems		
		Periquita	Gamay	Donzelinho Tinto	Periquita	Gamay	Donzelinho Tinto	Periquita	Gamay	Donzelinho Tinto
Phenolic acids										
Hydroxybenzoic acid										
Gallic acid (**1**)	5.35	0.24 (0.00)	0.31 (0.00)	0.30 (0.00)	-	-	-	-	-	-
Hydroxycinnamic acid										
Caftaric acid (**2**)	9.17	-	-	-	-	-	-	1.21 (0.15)	0.56 (0.01)	0.75 (0.04)
Coutaric acid (**3**)	12.52	-	-	-	-	-	-	0.28 (0.00)	nq	0.21 (0.01)
∑		0.24 (0.00)	0.31 (0.00)	0.30 (0.00)	-	-	-	1.49 (0.15)	0.56 (0.01)	0.96 (0.05)
Flavonoids										
Flavan-3-ols										
Catechin (**4**)	16.43	3.82 (0.15)	8.96 (0.05)	4.08 (0.03)	-	-	-	2.36 (0.02)	2.98 (0.11)	6.74 (0.05)
Epicatechin (**5**)	23.69	2.95 (0.04)	3.96 (0.03)	0.66 (0.05)	-	-	-	nq	1.48 (0.04)	-
Epicatechin-3-*O*-gallate (**6**)	37.86	nq	nq	nq	-	-	-	-	-	-
∑		6.77 (0.19)	12.92 (0.07)	4.74 (0.08)	-	-	-	2.36 (0.02)	4.46 (0.15)	6.74 (0.05)
Flavonols										
Quercetin-3-*O*-galactoside (**7**) + Quercetin-3-*O*-rutinoside (**8**) *	49.65	-	-	-	1.72 (0.01)	0.93 (0.01)	5.93 (0.10)	0.25 (0.01)	0.47 (0.01)	1.68 (0.04)
Quercetin-3-*O*-glucoside (**9**)	51.11	-	-	-	1.37 (0.01)	0.95 (0.02)	2.16 (0.04)	0.67 (0.02)	0.56 (0.00)	-
Kaempferol-3-*O*-glucoside (**11**)	55.41	-	-	-	7.58 (0.02)	3.37 (0.04)	7.89 (0.16)	-	-	-
∑		-	-	-	10.67 (0.04)	5.25 (0.07)	15.98 (0.30)	0.92 (0.03)	0.98 (0.01)	1.68 (0.04)
Anthocyanins										
Malvidin-3-*O*-glucoside (**10**)	51.61	-	-	-	39.13 (1.33)	42.59 (0.72)	41.28 (0.12)	0.83 (0.03)	0.68 (0.01)	nq
Total		7.01 (0.19)	13.23 (0.07)	5.04 (0.08)	49.80 (1.37)	47.84 (0.79)	57.26 (0.42)	5.60 (0.23)	6.68 (0.18)	9.38 (0.14)

Results correspond to mean (standard deviations) of three individual determinations. “-”: not detected; “nq”: not quantified; “∑”: sum of the identified phenolic compounds; * quercetin-3-*O*-galactoside and quercetin-3-*O*-rutinoside were quantified together.

**Table 2:** IC_50_ values (mg lyophilized extract/mL) obtained from red grape hydroethanolic extract.

Varieties	Extracts	DPPH Scavenging
Periquita	Seeds	0.43 ± 0.01
Gamay		0.23 ± 0.01
Donzelinho Tinto		0.36 ± 0.01
Periquita	Skins	0.72 ± 0.02
Gamay		0.90 ± 0.01
Donzelinho Tinto		0.98 ± 0.01
Periquita	Stems	0.50 ± 0.05
Gamay		0.50 ± 0.02
Donzelinho Tinto		0.41 ± 0.01

Values are expressed as the mean ± SD of three independent experiments performed in triplicate.

**Table 3:** Minimum inhibitory concentration (MIC) of red grape skin, stem, and seed extracts of three Portuguese grape varieties: Periquita, Gamay, and Donzelinho Tinto.

Bacteria Collection				MIC (μg/mL)					
	Seeds			Stems			Skins		
	Periquita	Gamay	Donzelinho Tinto	Periquita	Gamay	Donzelinho Tinto	Periquita	Gamay	Donzelinho Tinto
Gram-negative									
*E. coli* from pigs									
S1	10	10	10	25	10	50	-	-	-
S2	-	-	-	-	-	-	-	-	-
S3	-	-	-	-	-	-	-	-	-
S4	-	-	-	-	-	-	-	-	-
S17	-	-	-	-	-	-	-	-	-
S18	50	25	-	-	-	-	-	-	-
S21	-	-	-	-	-	-	-	-	-
S25	-	-	-	-	-	-	-	-	-
S31	-	-	-	-	-	-	-	-	-
S33	-	-	-	-	-	-	-	-	-
S34	-	-	-	-	-	75	-	-	-
S40	-	-	-	-	-	-	-	-	-
S42	-	-	-	-	-	-	-	-	-
*E. coli* from rabbits									
C1	-	-	-	-	-	-	-	-	-
C3	-	-	-	-	-	-	-	-	-
C5	-	-	-	-	-	-	-	-	-
C9	-	-	-	-	-	-	-	-	-
C18	-	-	-	-	-	-	-	-	-
C24	-	-	-	-	-	-	-	-	-
C30	-	-	-	-	-	-	-	-	-
C31	-	-	-	-	-	-	-	-	-
C33	-	-	-	-	-	-	-	-	-
C34	-	-	-	-	-	-	-	-	-
C36	-	-	-	-	-	-	-	-	-
C40	-	-	-	-	-	-	-	-	-
C48	-	-	-	-	-	-	-	-	-
Gram-positive									
*L. monocytogenes* from food products and associated environments									
L1	10	10	10	25	10	-	-	-	-
L2	-	-	-	10	25	-	-	-	-
L3	10	10	10	25	25	50	75	-	75
L4	10	25	25	25	-	10	75	-	75
L6	10	10	10	25	50	50	75	-	75
L7	10	10	10	10	25	10	75	-	75
L8	10	10	10	10	10	-	75	-	75
L10	10	10	10	25	-	-	-	-	-
L11	10	10	10	25	25	10	-	-	-
L12	10	10	10	25	50	-	-	-	-
L13	10	10	25	25	25	-	75	-	75
L14	25	10	25	10	-	100	-	-	-
L15	25	10	10	50	50	-	-	-	-

“-’’: not detected.

**Table 4:** Regression equation, *r*^2^ value s and linearity range of the reference compounds used for phenolics quantification.

Compound	Regression Equation (mg/mL)	*r* ^2^	Linearity (mg/mL)
Gallic acid	y = 4.20 × 10^4^x − 226.34	0.997	0.011–0.178
Caffeic acid	y = 9.20 × 10^4^x − 1370.40	0.998	0.066–1.060
p-Coumaric acid	y = 1.20 × 10^5^x − 286.89	0.998	0.008–0.567
Catechin	y = 1.12 × 10^4^x − 172.76	0.998	0.050–1.270
Epicatechin	y = 1.20 × 10^5^x + 85.90	0.986	0.018–1.140
Epigallocatechin-3-*O*-gallate	y = 2.90 × 10^4^x + 1218.40	0.993	0.063–1,000
Quercetin-3-*O*-rutinoside	y = 2.90 × 10^4^x + 39.21	0.997	0.016–0.194
Quercetin-3-*O*-glucoside	y = 4.80 × 10^4^x − 126.34	0.995	0.005–0.380
Kaempferol-3-*O*-glucoside	y = 3.70 × 10^3^x − 14.74	0.997	0.060–0.730
Malvidin-3-*O*-glucoside	y = 1.00 × 10^4^x + 127.23	0.992	0.062–1.000

## Data Availability

The original contributions presented in the study are included in the article; further inquiries can be directed to the corresponding authors.
